# Tracking progress in anthropometric failure among under-five children in Ethiopia: a geospatial and multilevel analysis

**DOI:** 10.1186/s13690-021-00615-2

**Published:** 2021-06-16

**Authors:** Binyam Tariku Seboka, Samuel Hailegebreal, Delelegn Emwodew Yehualashet, Abel Desalegn Demeke

**Affiliations:** 1 School of Public Health, Dilla University, Dilla, Ethiopia; 2 Department of Health Informatics, Arbaminch University, Arbaminch, Ethiopia; 3 Department of Nursing, Dilla University, Dilla, Ethiopia

**Keywords:** Anthropometric failure, undernutrition, Composite Index of Anthropometric Failure (CIAF), Children, Spatial analysis, Multilevel analysis, Ethiopia

## Abstract

**Background:**

Undernutrition is a major public health concern among under-five children in many developing countries. This work evaluated the overall prevalence of under-nutrition by using a composite index of anthropometric failure (CIAF), which helps in the detection of children with multiple anthropometric failures. This research also includes the Spatio-temporal distribution of childhood anthropometric failures across time.

**Methods:**

Secondary data was obtained from the Ethiopian Demographic and Health Survey for the survey 2005, 2011, and 2016 years. Data included 23,864 samples of children between the ages of 0–59 months, which is a nationally representative sample in Ethiopia. Other than descriptive statistics, the multivariate multilevel logistic regression was used to identify associated factors, and Getis-Ord spatial statistical tools were employed to identify high and low hotspots areas of anthropometric failures.

**Result:**

The prevalence obtained with CIAF in 2005, 2011, and 2016 was, 53.5 %, 51 %, and 46.2 % of children were suffering from under-nutrition respectively. The spatial analysis revealed areas that are at a higher risk of anthropometric failures consistently were found in northern parts of the country, largely in the Amhara, Tigray, and Afar regions. Multilevel logistic regression analysis showed that the risk of anthropometric failure was higher among older children, had low birth weight, had a mother with low BMI, was in a rural area, had mothers and fathers without formal education.

**Conclusions:**

In addition to identifying wasted, stunted, and underweight children, CIAF also identified children with multiple conditions, which are often overlooked in nutritional surveys. As revealed by this composite index, the prevalence of anthropometric failure remains considerably high and its spatial distribution also significantly varied across the regions in the country. The established socio-demographic characteristics and districts with a higher risk of anthropometric failure can be used to develop localized intervention and prevention strategies to improve Ethiopian children’s nutritional status and healthcare.

## Background

Nutrition is to be understood as both an input to and an outcome of sustainable development [1]. Malnutrition includes several forms of under-nutrition as well as overweight and obesity. Under-nutrition in a population of under-five children is commonly measured by three anthropometric indicators, namely stunting (low height for age), wasting (low weight for height), and underweight (low weight for age). Acute and chronic nutritional deficiencies are reflected in stunting and wasting, respectively. in addition, being underweight reflect both acute and chronic nutritional deficiency [1, 2]. As a result, anthropometric deficiencies have both short-term and long-term negative effects. In the short term, it leads to ill health and mortality among children. In the long-term, it leads to impaired cognitive development, poorer educational achievement, and impeded economic productivity [1, 3].

Since antiquity, undernutrition has been a public health problem for mankind, and it continues to be so today, posing a significant challenge to global public health. It is the leading cause of morbidity and mortality in children under the age of five worldwide, with Africa, Central Asia, and Southern Asia being the most affected areas [4, 5]. Ethiopia alone accounted for over 178 thousand under-five deaths in Sub-Saharan Africa in 2016 [4, 6], with anthropometric failures contributing significantly to this figure [7, 8]. Understanding the risk factors for undernutrition is critical for policymakers to establish targeted prevention and intervention strategies for reducing the number of undernourished children, given the severe implications of undernutrition.

Over a decade, the prevalence of underweight, stunted, and wasted children in Ethiopia has decreased, according to estimates from the Demographic and Health surveys(DHS) [9–11]. Despite these downward trends, under-nutrition continues to be a significant problem. Furthermore, most studies that examined the prevalence of under-nutrition focused on single conventional measures like wasting, stunting, and underweight in children under the age of five [12]. However, studies have shown that using conventional metrics alone does not provide accurate estimates of the true burden of childhood under-nutrition [13]. This is because, these metrics may overlap, a single child may exhibit symptoms of two or more of them.

Previous studies have identified factors that are linked to an increased risk of undernutrition in children under the age of five around the world. The age of the women at birth, marital status, education level, employment status, and the number of children in the household are some of the determinants [14–16]. Moreover, evidence indicated that poor socioeconomic conditions, food insecurity, poverty, and adverse conditions such as illness or inadequate feeding practices can drastically alter the growth pattern of children [14, 16–18].

Children’s, nutritional status is influenced by various factors including the environment, communities, and socioeconomic condition of the area where they lived. Furthermore, anthropometric failures are considered to be not uniformly distributed in space [2, 19]. Due to the unequal distribution of anthropometric failure risks, ignoring this correlation may lead to incorrect inference. Despite the numerous spatial studies published in the literature, only a few attempts have been made to use spatial analysis techniques to estimate the overall geographic trends of childhood anthropometric failure. This research aims to add to the body of knowledge by identifying the high-risk areas of childhood anthropometric failures, which can be useful in developing localized strategies and guiding policymakers in developing successful public health interventions to minimize undernutrition-related morbidity and mortality in children.

This study aimed to assess the prevalence of childhood anthropometric failures in Ethiopia using spatial and spatial-temporal analysis based on secondary data obtained from three previous Ethiopian demographic health surveys (EDHS). Furthermore, assessed the relationship of demographic, socioeconomic, and environmental determinants of anthropometric failures among under-five children.

## Method

### Study design, setting, and period

The survey-based cross-sectional study design was employed by using data that have been taken from the last three rounds of Ethiopian demographic and health survey (EDHS) 2005, 2011, and 2016 [9–11]. These are nationally representative surveys conducted in Ethiopia, which is located in the horn of Africa (3^°^-14^°^N and 33^°^– 48^°^E). Ethiopia is divided into nine administrative regions and two administrative cities, with each region, is sub-divided into zones, districts, towns, and kebeles (the smallest administrative units). The DHS used a multistage stratified cluster sampling method in which sample households were chosen within clusters EAs (enumeration areas). Children aged 0–59 months were measured for height and weight In the selected households [11].

Accordingly, a total of 23,864 children under the age of five were screened for their anthropometry status. Of these, 4,130 were screened in 2005, 10,285 in 2011, and 9,449 in 2016. The detailed sampling procedure was presented in the full EDHS report [9–11]. The geographic coordinate (longitude and latitude) data were taken from the selected EAs.

DHS granted permission to access the EDHSs data sets and the geographic coordinate data through the project title “Tracking Progress in Anthropometric Failure among Under-Five Children in Ethiopia: A Geospatial Analysis”. The survey data was extracted by using STATA version 14.1, the locational data (latitude and longitude) were extracted from the downloaded excel file. Then after extracting locational data for each survey, DHS clusters with missing values of the locational data (latitude and longitude) were excluded from the study, and the spatial analysis was conducted with a complete set of locational data.

### Measurement of variables

#### Dependent variables

All children younger than five years of age were selected for anthropometry measurements. First, the classification of children as stunted, wasted, and underweight were conducted based on their Z-scores for weight-for-height (WHZ), height-for-age (HAZ), weight-for-age (WAZ), according to the World Health Organization (WHO) guideline. Stunting (chronic malnutrition) is defined as a Height for Age Z-score (HAZ) of < -2. Wasting (acute malnutrition) is defined as a Weight for Height Z-score (WHZ) of < -2. Underweight (mixed acute and chronic malnutrition) is defined as Weight for Age Z-score (WAZ) of <-2 [4, 20].

Second, based on the above classification of children to various categories of undernutrition, an estimate of the overall prevalence of under-nutrition as a single measure among children under 5 years was measured using CIAF. Second, using CIAF, an estimation of the overall prevalence of undernutrition as a single measure among children under the age of five years was calculated based on the above classification of children into different categories of undernutrition.

CIAF is based on a cross-classification scheme, involving the categories obtained by applying the cut-off ± 2 standard deviation to the conventional indices underweight (weight-for-age), stunting (height-for-age), and wasting (weight-for-height). Children nutritional indicators were categorized into seven groups: (A) no failure; (B) wasting only; (C) wasting and underweight; (D) wasting, stunting, and underweight; (E) stunting and underweight; (F) stunting only; and (Y) underweight only. Therefore, a child is considered as undernourished, as measured in CIAF, if he or she is suffering from any anthropometric failure (B–Y) above [13].

#### Independent variables

Factors that have been associated with child under-nutrition based on the UNICEF general conceptual framework for causes of malnutrition [21] and a review of prior similar studies were included in the study [14, 16, 18, 22]. A series of information was extracted from three round of EDHSs including socio-demographic and economic characteristics: sex, age of the child, educational level of the mother, maternal age at first birth, place of residence, wealth index, place of delivery, maternal marital status, Body Mass Index (BMI) of the mother, ANC(Antenatal Care) visit, time of initiation of breastfeeding, maternal employment status, paternal employment status, number of children born, birth order, preceding birth interval, and symptom of fever, cough, and diarrhea in the child.

In the analyses, age was recorded as 0–5, 6–11, 12–23, 24–35, and, 36 above months. The mother’s BMI was determined by dividing her weight in kilograms by her height in meters squared (kg/m2). Mothers were categorized according to three risk factors, “underweight” was defined as a BMI of less than 18.5 k/m2, “normal” was defined as a BMI of 18.5-24.95 k/m2, and “overweight/obese” was defined as a BMI of more than 25-29.9 kg/m2. The wealth index was divided into quintiles, and each household was assigned its corresponding category (1 being “Poorest” and 5 “Richest” in wealth). Birth order is the number of children a woman has; we have categorized it as “1–2”,” 3–4”, “5 or more”. Maternal and paternal education levels were recoded into three, “no education”, “primary”, and “secondary and above education”.

### Data management and analysis

The data were cleaned using STATA 14.1 software and Microsoft excel. The data were weighted using sampling weight, primary sampling unit, and strata before any statistical analysis to restore the representativeness of the survey and to take into account sampling design when calculating standard errors to get reliable statistical estimates. Before any statistical analysis, the data were weighted using sampling weight, primary sampling unit, and strata to restore the survey’s representativeness and to account for sampling design when calculating standard errors to obtain reliable statistical estimates.

Cross tabulations and summary statistics were conducted to describe the study population. Descriptive and summary statistics were conducted using STATA version 14.1, ArcGIS version 10.8, and SaTScan version 9.6 software.

#### Spatial analysis

ArcGIS version 10.8 software and SaTScan version 9.6 software were used to explore the Spatio-temporal distribution of anthropometric failure. The global spatial autocorrelation (Global Moran’s I) was done to assess whether childhood anthropometric failures were dispersed, clustered, or randomly distributed in the study area. Moran’s I is a correlation coefficient that measures the overall spatial autocorrelation of a data set. It is one way to test for autocorrelation and has a value from − 1 to 1. A Moran’s value of -1 is perfect clustering of dissimilar values (this can be called perfect dispersion), a value of 0 is no autocorrelation (perfect randomness), and a value of + 1 indicates perfect clustering of similar values (it’s the opposite of dispersion). A statistically significant Moran’s I (*p* < 0.05) showed that childhood anthropometric failure is non-random.

Getis-OrdGi* statistics were computed to measure how spatial autocorrelation varies over the study location by calculating GI* statistic for each area. Z-score is computed to determine the statistical significance of clustering, and the p-value was computed for the significance. Statistical output with high GI* indicates “hotspot” whereas low GI* means a “cold spot”. Hot spot areas indicated that there was a high proportion of anthropometric failure and the cold spot areas indicated that there was a low proportion of anthropometric failure.

Furthermore, the spatial interpolation technique was used to predict childhood anthropometric failure on the un-sampled areas in the country based on the value observed from sampled EAs. The Kriging interpolation method was employed to explore the burdens of childhood anthropometric failure in the un-sampled areas of the country based on the observed data since it had low mean square error and residual as compared to the other interpolation techniques [23].

#### Cluster analysis

Spatial scan statistical analysis of Bernoulli-based model was employed to test for the presence of statistically significant spatial clusters of anthropometric failure using Kulldorff’s Sat Scan version 9.6 software. The spatial scan statistic uses a circular scanning window that moves across the study area. Children with anthropometric failure were taken as cases and those who were free from anthropometric failure as controls to fit the Bernoulli model. The spatial cluster size of < 25 % of the population was used, to include sparsely populated regions that may have similar risk factor profiles. The scanning window with maximum likelihood was the most likely performing cluster, and the p-value was assigned to each cluster using Monte Carlo hypothesis testing by comparing the rank of the maximum likelihood from the real data with the maximum likelihood from the random datasets. The primary and secondary clusters were identified and assigned p-values and ranked based on their likelihood ratio test, based on 999 Monte Carlo replications [24].

#### Multilevel logistic regression

Multivariable multilevel logistic regression was used to analyze factors associated with childhood stunting at two levels: individual and community (cluster) levels. Four models were fitted using STATA command melogit and xtmelogit. The first model without any explanatory variables evaluates the extent of the cluster variation on anthropometric failure. The second model with only individual-level variables, the third model with only community-level variables, and the fourth model both individual-level and community-level variables. A *P*-value of < 0.05 was used to define statistical significance. Adjusted Odds Ratios (AOR) with their corresponding 95 % confidence intervals (CIs) were calculated to identify the independent predictors of anthropometric failure. Model comparison was conducted by using deviance and likelihood ratio.

The intra-cluster correlation coefficient (ICC), Median Odds Ratio (MOR), and proportional change in variance (PCV) statistics were calculated to measure the variation between clusters. ICC was used to explain cluster variation while MOR is a measure of unexplained cluster heterogeneity. ICC quantifies the degree of heterogeneity of childhood anthropometric failure between clusters (the proportion of the total observed individual variation in anthropometric failure that is attributable between cluster variations) but MOR is quantifying the variation or heterogeneity in outcomes between clusters and is defined as the median value of the odds ratio between the cluster at high risk of anthropometric failure and cluster at low risk when randomly picking out two clusters (EAs) MOR. PCV measures the total variation attributed by individual-level factors and community-level factors in the multilevel model as compared to the null model PCV [25].

## Result

This report includes data from 4130 children’s,10,285 children, and 9449 children from DHS surveys conducted in 2005, 2011, and 2016 respectively. The estimated prevalence of anthropometric failures by CIAF and conventional indicators are shown in Table 1. Over the last decade, there has been a decline in the magnitude of anthropometric failures as Table 1 shows, the overall proportion of anthropometric failures among under-five children has been decreased from 53.5 % (95 % CI: 52.3, 55.5) in 2005 to 46.2 % (95 % CI: 45.5, 47.6) in 2016. When compared to conventional anthropometric indices CIAF identified more children who had one form of anthropometric failures, while conventional anthropometric indices underestimate the number of children with the nutritional problem (Table 2).

**Table 1:** Classification of children with CIAF and conventional indicators in Ethiopia (2005–2016)

Conventional categories	EDHS 2005	EDHS 2011	EDHS 2016
	**n = 4130**	**n = 10,285**	**n = 9449**
Wasting	484(10.5)	1052(9.7)	1033(10)
Stunting	2133(46.5)	4834(44.4)	3980(38.4)
Underweight	1761(38.4)	3122(28.7)	2488(24)
CIAF Total	2211(53.5)	5249(51)	4364(46.2)

### Trends of anthropometric failure

**Fig. 1:**
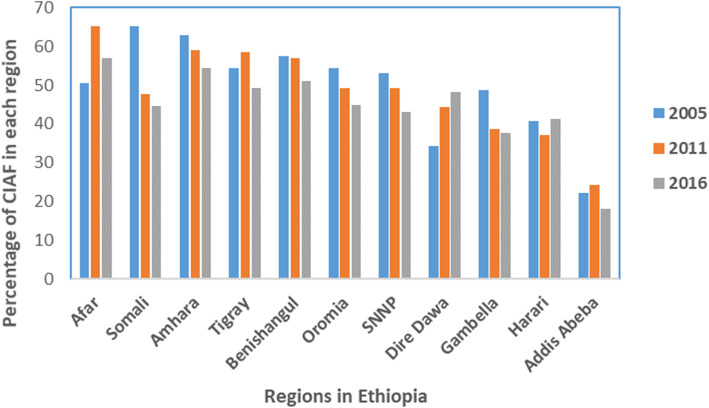
Trends of anthropometric failure over time across regions in Ethiopia, EDHS 2005–2016

Table 3 shows the background characteristics of participants and the result of the chi-square test of association with anthropometric status. The result indicated the prevalence and trends in anthropometric failures among under-five children varied by child and maternal background characteristics. Among background characteristics considered, wealth index, place of residence, mothers BMI status, place of delivery, and childhood diarrhea were significantly associated with anthropometric failures over three surveys. Across all surveys, the prevalence of anthropometric failures tended to be higher in children presented with diarrhea.; about 50.1 %, 56 %, and 49.4 % of children with a history of diarrhea two weeks before the survey had anthropometric failures in 2005, 2011, and 2016 respectively. Among children who were born at home, 57 % in 2005, 54 % in 2011, and 51 % in 2016 had anthropometric failures. There was a significant association between maternal BMI and children anthropometric failure; among children who had thin mothers, 58.3 %, 57 %, and 54 % in the respective survey years had anthropometric failures. Similarly, the wealth pattern of households shows that children from the poorest wealth quintile show the highest prevalence of anthropometric failures. Overall, a greater proportion of children’s had anthropometric failures in rural areas (53.6) than in urban areas (36 %). Further, the prevalence of childhood anthropometric failures shows a variation across regions; in 2016, for example, the range was from 56.9 % in the Afar region to 18.02 % in the Addis Ababa region (Fig. 1).

**Table 2:** Distribution of children under five years according to the seven categories of CIAF classification of nutritional status, based on data obtained from three surveys (2005–2016) conducted in Ethiopia

**CIAF categories**		**EDHS 2005**	**EDHS 2011**	**EDHS 2016**
		*n*=4130	*n*=10285	*n*=9449
		*n* (%)	*n* (%)	*n* (%)
A	No failure	1919(46.5)	5036(49)	5085(53.74)
B	Wasting only	98(2.4)	340(3.31)	398(4.21)
C	Wasting + underweight	182(4.4)	391(3.8)	362(3.83)
D	Wasting + stunting + underweight	167(4)	460(4.4)	357(3.77)
E	Stunting + underweight	1023(24.7)	2083(20.2)	1477(15.61)
F	Stunting only	585(14.2)	1836(17.8)	1606(16.9)
Y	Underweight only	156(3.8)	139(1.4)	164(1.7)
**CIAF (%)**		2211(53.5)	5249(51)	4364(46.2)
**Total (%)**		100	100	100

**Table 3:** Bivariate analysis showing the proportion of children age 6–59 months who had anthropometric failures by a child and maternal background characteristics, EDHS 2005, 2011, 2016

Variables	2005 EDHS		2011 EDHS		2016 EDHS		Pooled data	
	*p*-value/ **percent**	*n*	*p*-value/ **percent**	*n*	*p*-value/ **percent**	*n*	*p*-value/ **percent**	*n*
**Sex of child**	0.175		0.000		0.000			
Male	54.99	1,075	52.79	2,539	47.9	2137	51.9	5751
Female	52.81	997	48.87	2,269	44.0	1890	48.5	5156
**Age of child**	0.000		0.000		0.000			
0-5 m	16.51	54	26.67	264	27.6	252	23.6	570
6-11 m	41.86	162	34.28	327	31.5	300	35.9	789
12-23 m	62.77	472	52.87	931	47.4	842	54.3	2245
24-35 m	59.33	445	60.67	1,120	55.2	937	58.4	2502
36 and above	57.71	939	55.51	2,166	49.6	1696	54.3	4801
**Had cough in last two weeks**	0.033		0.100		0.088			
Yes	50.08	1,753	52.53	1,018	48.0	696	50.2	3467
No	54.68	319 |	50.43	3,790	45.6	3331	50.2	7440
**Had fever in last two weeks**	0.727		0.000		0.008			
Yes	53.32	377 |	54.62	1,028	49.4	620	52.4	2025
No	54.05	1,695	49.93	3,780	45.4	3047	49.8	8522
**Had diarrhea recently**	0.033		0.000		0.021			
Yes	50.08	319	55.74	820	49.4	497	51.7	1636
No	54.68	1,753	49.96	3,988	45.6	3530	50.1	9271
**Birth weight**	0.037		0.002		0.000			
Low < 2500	40.00	12	36.27	37	46.0	93	40.8	142
Normal > = 2500	22.59	54	22.60	174	30.4	480	25.2	708
**Age of mother at first birth**	0.117		0.000		0.000			
< 20	54.95	1,549	52.19	3,560	47.8	3018	51.6	8127
20–34	51.18	520	47.38	1,232	41.4	1004	46.7	2756
35–49	50.00	2	62.50	10	25.0	5	45.8	17
**Breastfeeding Initiation**	0.080		0.031		0.155			
Within an hour	53.92	957	48.46	1,720	43.8	1890	48.7	4567
After an hour	47.75	191	50.27	835	46.8	552	48.3	1578
After one day	52.00	182	52.73	657	195	42.9	49.2	1034
**Place of delivery**	0.000		0.000		0.000			
Health facilities	28.80	106	31.39	382	35.8	995	31.9	1483
At home	56.60	1,937	53.67	4,362	50.8	2981	53.7	9280
Other places	57.14	28	62.37	58	49.5	51	56.3	137
**Birth order**	0.000		0.000		0.000			
1st	48.53	627	46.43	1,579	42.3	1367	45.8	3573
3–4	55.90	597	53.00	1,380	45.3	1089	51.4	3066
5 and more	57.27	847 |	53.70	1,843	50.4	1571	53.8	4261
**Residence**	0.000		0.000		0.000			
urban	32.58	172	32.94	499	31.8	512	36.0	1183
Rural	57.35	1,899	54.32	4,303	49.2	3515	53.6	9717
**Household size**	0.003		0.013		0.000			
1–4	50.56	448	48.87	1,123	42.5	1008	47.3	2579
5–9	55.69	1,493	51.93	3,317	47.2	2752	51.6	1562
10 and more	47.79	130	48.20	362	48.5	267	48.1	759
**Mothers BMI**	0.000		0.000		0.000			
Thin	58.27	511	56.99	1,410	53.7	1103	56.3	3024
Normal	53.87	1,490	50.38	3,206	45.9	2664	50.1	7360
Overweight	35.71	70	31.05	186	26.3	215	31.0	471
**Mother educational level**	0.000		0.000		0.000			
No education	58.15	1,705	54.76	3,598	50.83	232	54.6	5535
Primary	47.36	296	45.67	1,097	42.43	2,834	45.0	1409
Secondary and above	24.82	70	23.06	107	25.33	961	24.4	379
**Wealth index**	0.000		0.000		0.000			
Poorest	60.51	599	57.70	1,679	53.6	1682	57.3	3960
Poorer	59.83	429	56.89	995	51.8	794	56.2	2218
Middle	56.91	391	52.35	814	46.6	588	52.0	1793
Richer	53.19	358	49.58	772	40.4	446	47.7	1576
Richest	38.08	294 |	32.53	542	30.0	517	33.5	1353
**Current marital status**	0.424		0.095		0.895			
Currently not in union	56.43	136	54.08	345	46.3	224	52.3	705
Currently in union	53.78	1,935	50.65	457	45.9	3803	50.1	6195
**Preceding birth interval**	0.001		0.000		0.000			
≤ 24 months	60.88	445	56.02	1,014	51.4	892	56.1	2351
> 24 months	54.05	1,320	50.72	2,956	45.5	2407	50.01	6683
**ANC visit**	0.000		0.000		0.000			
Yes	44.04	377	42.31	1,226	41.4	1690	42.6	3293
No	57.09	962	55.61	1,993	50.1	1010	54.3	3965
**Source of drinking water**	0.283		0.000		0.000			
unimproved source	49.14	114	54.83	2,555	49.9	1710	51.3	4379
Improved source	52.84	1,154	47.04	2,232	43.5	2314	47.8	5700
**No of children under five years in the household**	0.225		0.012		0.000			
1	51.02	425	51.05	1,556	42.7	1313	48.3	3321
2	54.38	633	51.94	2,314	49.4	1929	51.9	4876
3	50.60	211	47.84	876 |	43.9	755	47.4	1842
**Husband/partner’s education**	0.000		0.000		0.000			
No education	58.43	821	56.45	2,749	52.02	2,069	56.6	5639
Primary	52.55	309	48.74	1,702	43.48	1,197	48.3	3208
Secondary and above	31.34	139	31.78	321	34.17	505	32.4	965

### The spatial trend of childhood anthropometric failure in Ethiopia

**Fig. 2:**
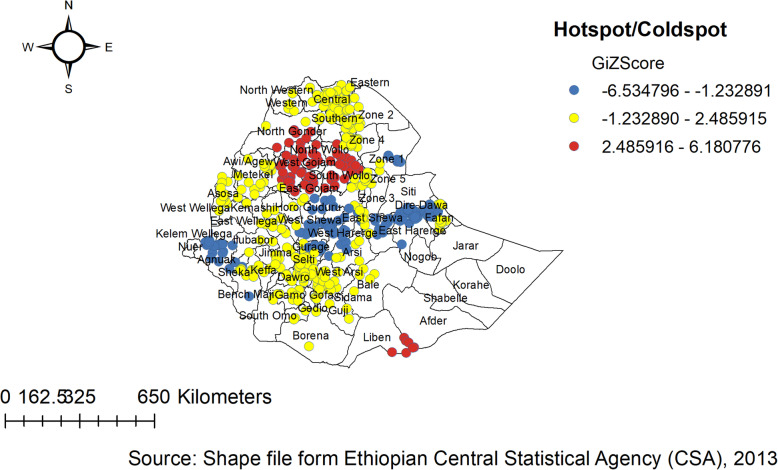
Hotspot and cold spot analysis of anthropometric failure in Ethiopia, EDHS 2005

**Fig. 3:**
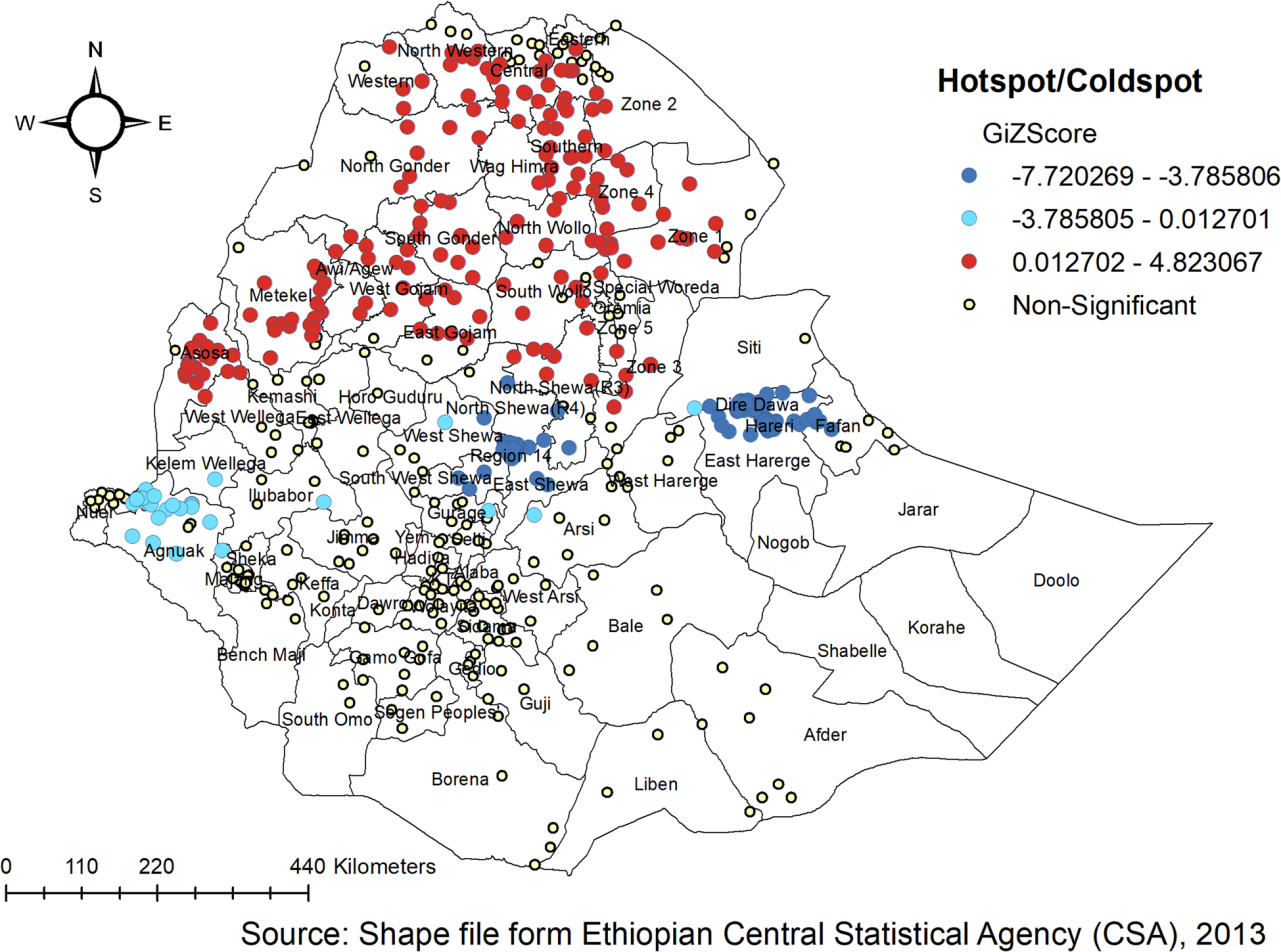
Hotspot and cold spot analysis of anthropometric failure in Ethiopia, EDHS 2011

**Fig. 4:**
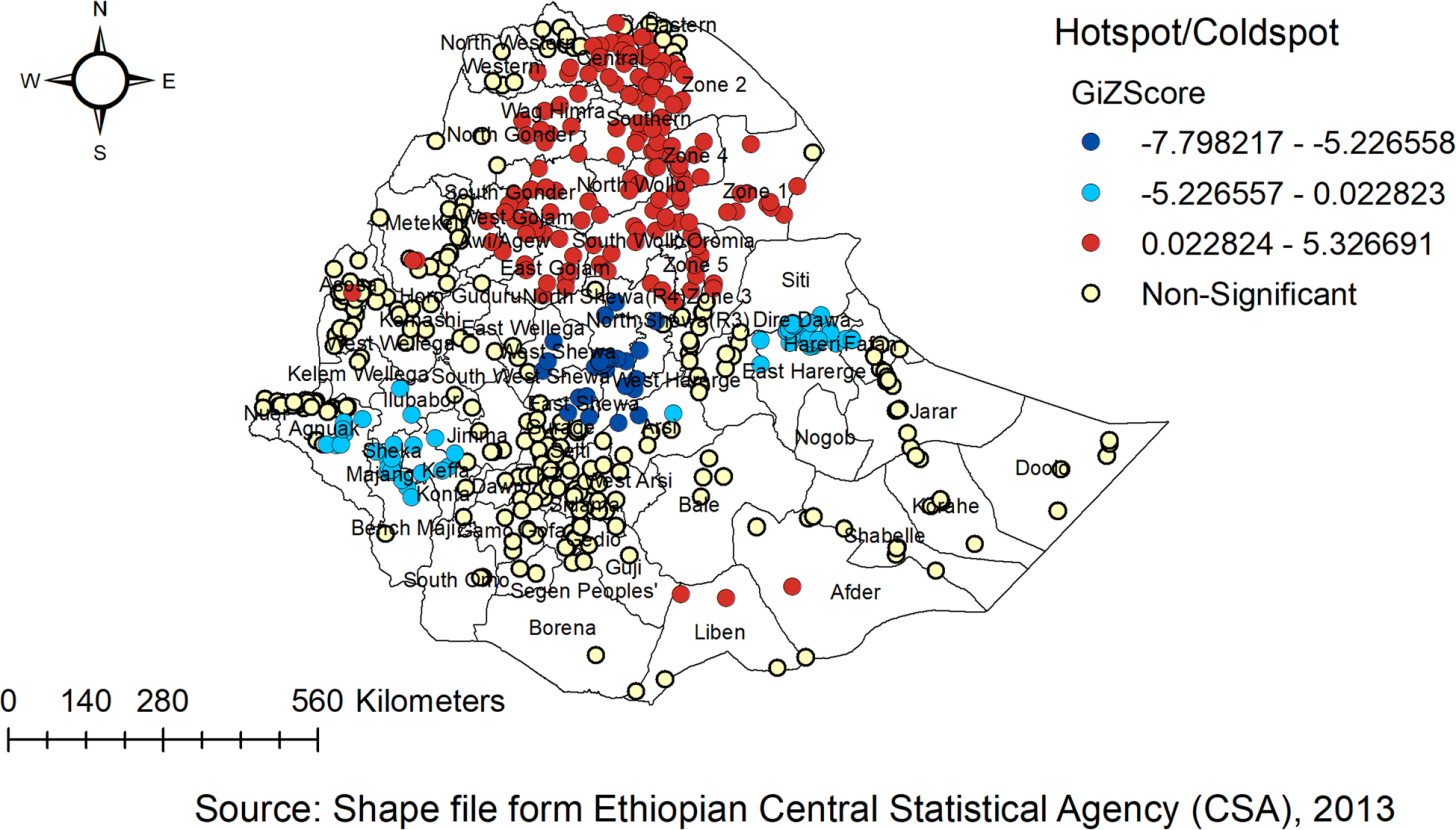
Hotspot and cold spot analysis of anthropometric failure in Ethiopia, EDHS 2016

The spatial autocorrelation analysis revealed the presence of statistically significant clusters at a 0.01, level of significance in each survey (Table 4). Graphical representations of the changes in the distribution of children’s anthropometric status by survey and districts are shown in the Figure. The spatial analysis indicates there were regional variations, with Tigray, Amhara, Afar, and Benishangul-Gumuz regions having a higher proportion of anthropometric failures, while the lowest proportion of anthropometric failures was observed in Addis Ababa and Gambella Regions consistently over time (Figs. 2, 3 and 4).

In the 2005 EDHS, almost all parts of Addis Ababa, Harari, and Dire Dawa were associated with a significantly low prevalence of childhood anthropometric failures (negative Z-score and Gipvalue < 0.05). Whereas, three zones in the Oromia region (Jimma, Horo Guduru, and West arsi), parts of Somali region (Liben, Afder, and fafan), four zones in the SNNP region (Yem, Woliyta, Dawro, and Sidama ), several zones of Amhara region (Awi/agew, Oromia, North Shewa, North Gonder, South Gondar, West Gojjam, East Gojjam, North wollo, and South wollo ), and Southern part of Tigray region were associated with a significantly high prevalence of childhood anthropometric failures (Positive Z-score and Gipvalue < 0.05). The notable spatial variations in the remaining zones were not significant (Fig. 2).

**Table 4:** Spatial autocorrelation analysis of anthropometric failure from EDHS (2005, 2011, and 2016)

**Survey**	**Pick Clustering distance in meters**	**Observed Moran’s***I*	**Expected Moran’s***I*	**Z-Score**	*P***-value**
**EDHS, 2005**	133994.0909	0.494	-0.001919	14.49	<0.01
**EDHS, 2011**	95786.7875	0.9102	-0.001754	28.79	<0.01
**EDHS, 2016**	121812.8929	0.4474	-0.00094	14.63	<0.01

Figure 3 shows the spatial variation of childhood anthropometric failures at the zonal level in 2011 EDHS. The spatial analysis shows that a statistically significant high proportion of anthropometric failures was found in northern parts of the country (Amhara, Benishangul-Gumuz, Tigray, and Afar regions), whereas statistically significant low spots of anthropometric failures were found in the western (Gambella), central Oromia, Addis Ababa and eastern parts of the country.

In the 2016 EDHS, hot spot areas for childhood anthropometric failures include six zones in the Tigray region (North-Western, Western, Southern, Eastern, Central, and East). North Gondar, South Gondar, West Gojam, East Gojam, awi/agew, North and South wollo of Amhara region. All zone of Afar region, Metekel, and Asosa of Benshangul Gumuz region, and Liben and Afder of Somali region (Fig. 4).

### Spatial scan statistics

**Fig. 5:**
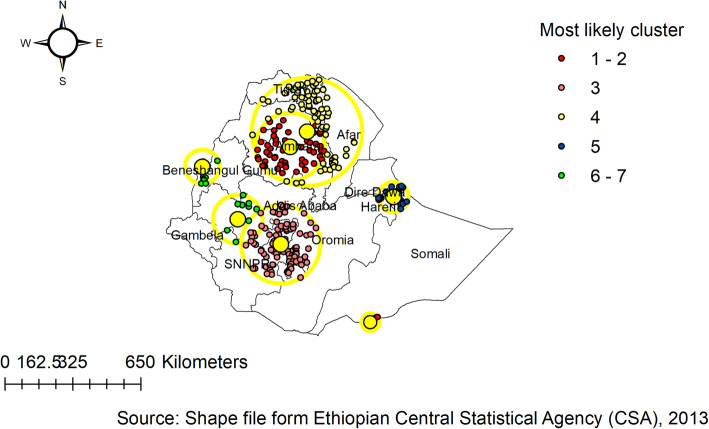
SaTScan scan statistics analysis of anthropometric failure in Ethiopia, EDHS 2005

In the 2005 EDHS, the spatial scan statistics identified a total of 6 significant clusters of anthropometric failures. Of these, one was most likely (primary cluster), the spatial window was located in Amhara and southern part Tigray regions centered at (11.529743 N, 38.342559 E) with 162.07 -km radius, a Relative Risk (RR) of 1.40, and Log-Likelihood (LLR) of 40.6, at *p*-value < 0.01 which was detected as the most likely cluster with Maximum Likelihood. It showed that children within the spatial window had 1.4 times more likely a higher risk of anthropometric failures than the children outside areas of the spatial window. Whereas the secondary clusters were located in the SNNP region, the southern part of Oromia, and the southern part of the Somali region (Fig. 5).

**Fig. 6:**
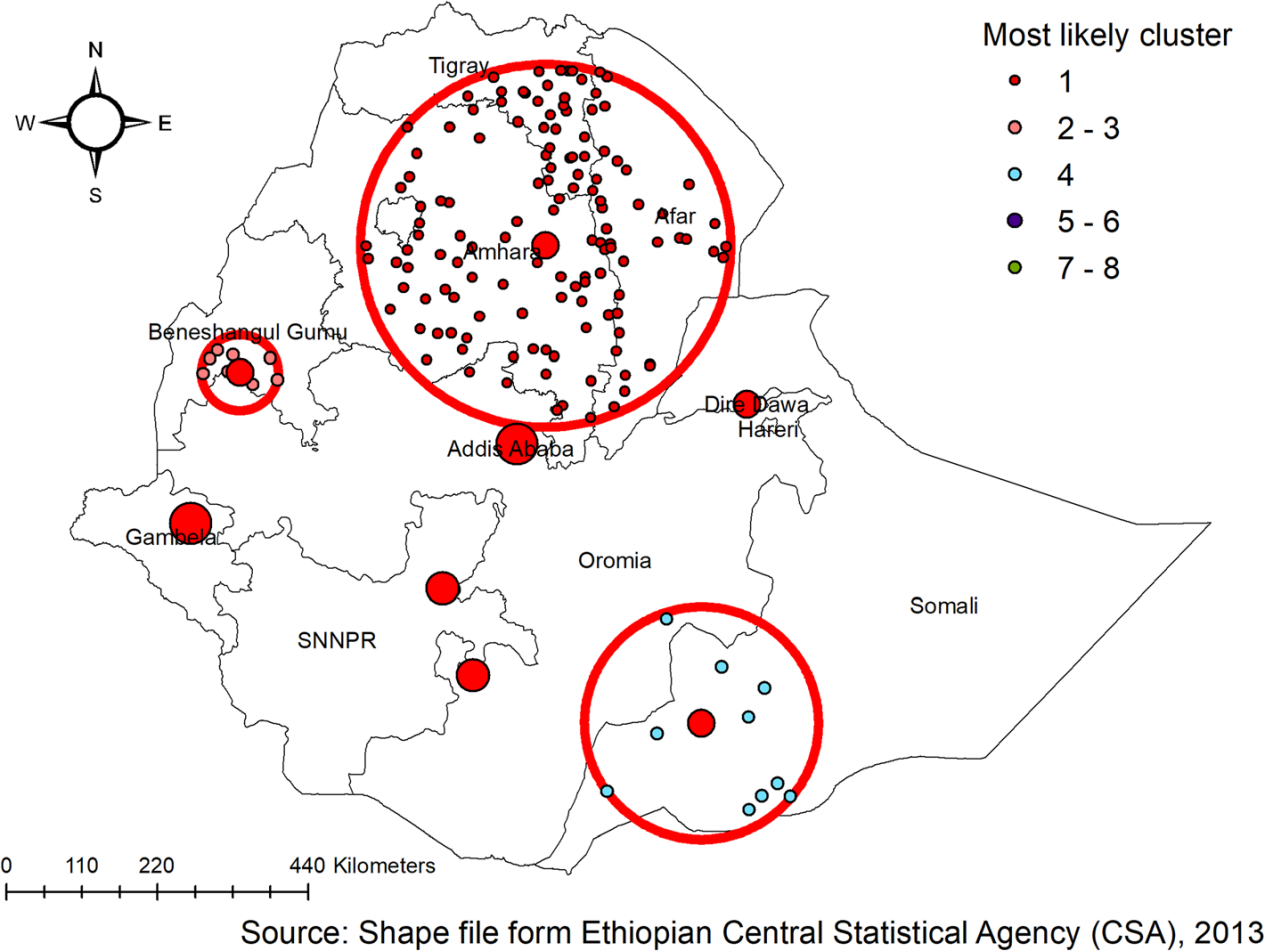
SaTScan scan statistics analysis of anthropometric failure in Ethiopia, EDHS 2011

In EDHS 2011, the spatial scan statistics identified a total of 3 significant clusters of anthropometric failures. Of these, one was most likely (primary cluster), the spatial window was located Amhara, Tigray, and afar regions centered at (11.646140 N, 39.234715 E) with 264.4-km radius, a Relative Risk (RR) of 1.3, and Log-Likelihood (LLR) of 75.8, at *p*-value < 0.01 which was detected as the most likely cluster with Maximum Likelihood. It showed that children within the spatial window had a 1.3 times higher likelihood of anthropometric failure as compared to children outside the spatial window. Whereas, the secondary clusters were located in the Benishangul region, which was centered at (9.981136 N, 35.224095 E) with a 55.24-km radius and LLR of 12.2, at *p*-value < 0.003. It showed that children within the spatial window had a 1.37 times higher risk of anthropometric failures than children outside the window (Fig. 6).

**Fig. 7:**
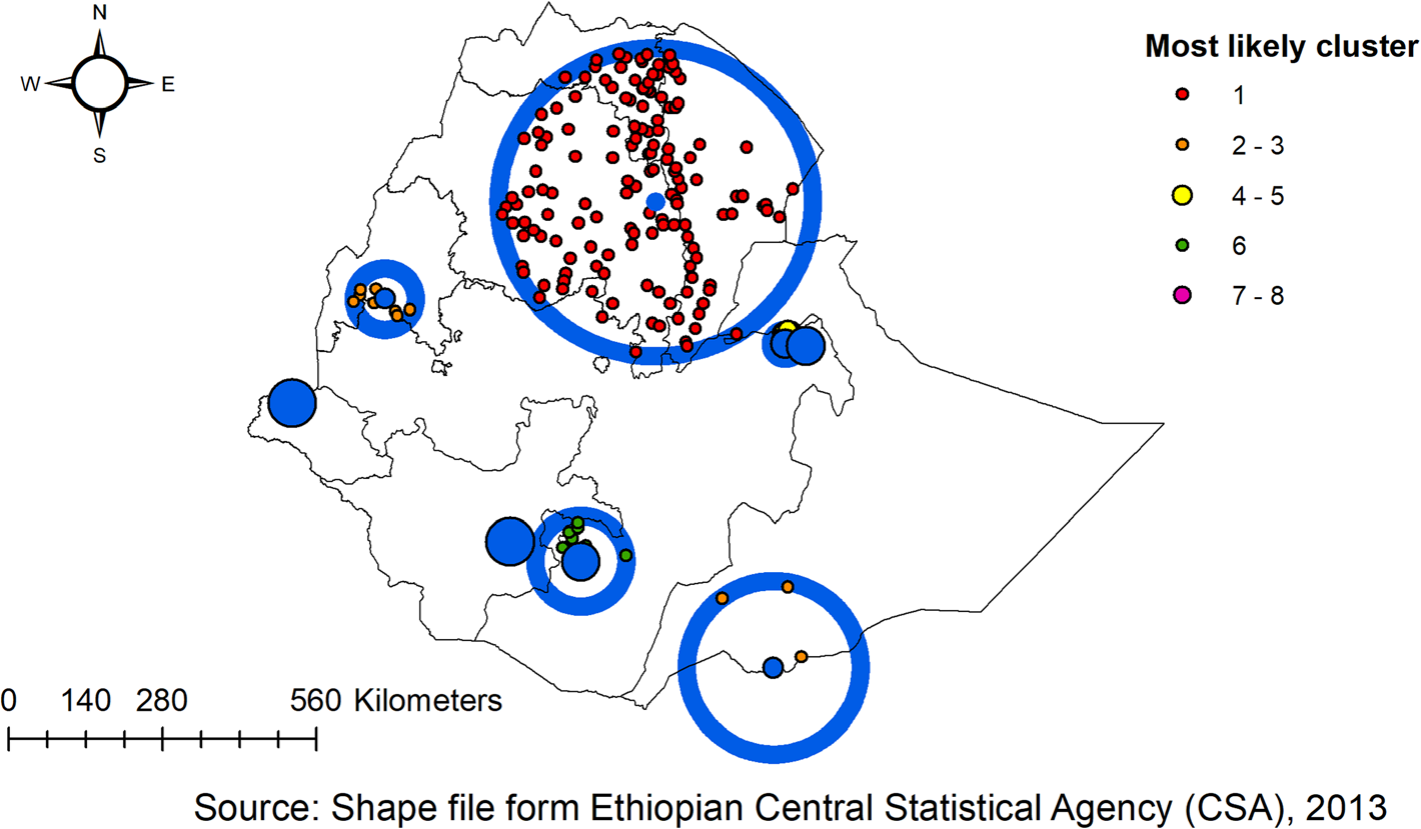
SaTScan scan statistics analysis of anthropometric failure in Ethiopia, EDHS 2016

In the 2016 EDHS, a total of 8 clusters were identified and four of them were significant clusters with *p*-value < 0.05. A total of 1236 locations/spots with a total sampled population of 2226 were found as primary cluster areas by using sat scan analysis with a *p*-value < 0.001. The primary cluster spatial window was located mainly in the Amhara, Tigray, and Afar regions centered at (11.626646 N, 39.666951 E) with 280.04 km radius, a Relative Risk (RR) of 1.3, and Log-Likelihood (LLR) of 58.48, at a *p*-value < 0.001 which was detected as the most likely cluster with Maximum Likelihood. It showed that children within the spatial window had a 1.31 times higher likelihood of anthropometric failures as compared to children outside of the spatial window. Whereas the secondary clusters were located in Benishangul and the southern part of Somali regions (Fig. 7).

### Spatial interpolation

**Fig. 8:**
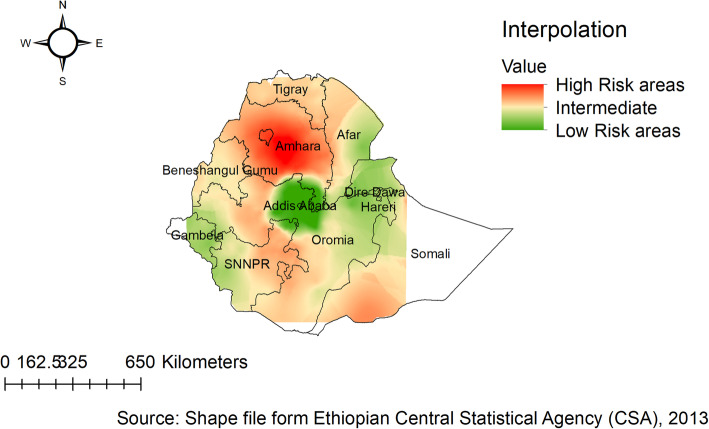
Ordinary Kriging interpolation of anthropometric failure in Ethiopia, EDHS 2005

**Fig. 9:**
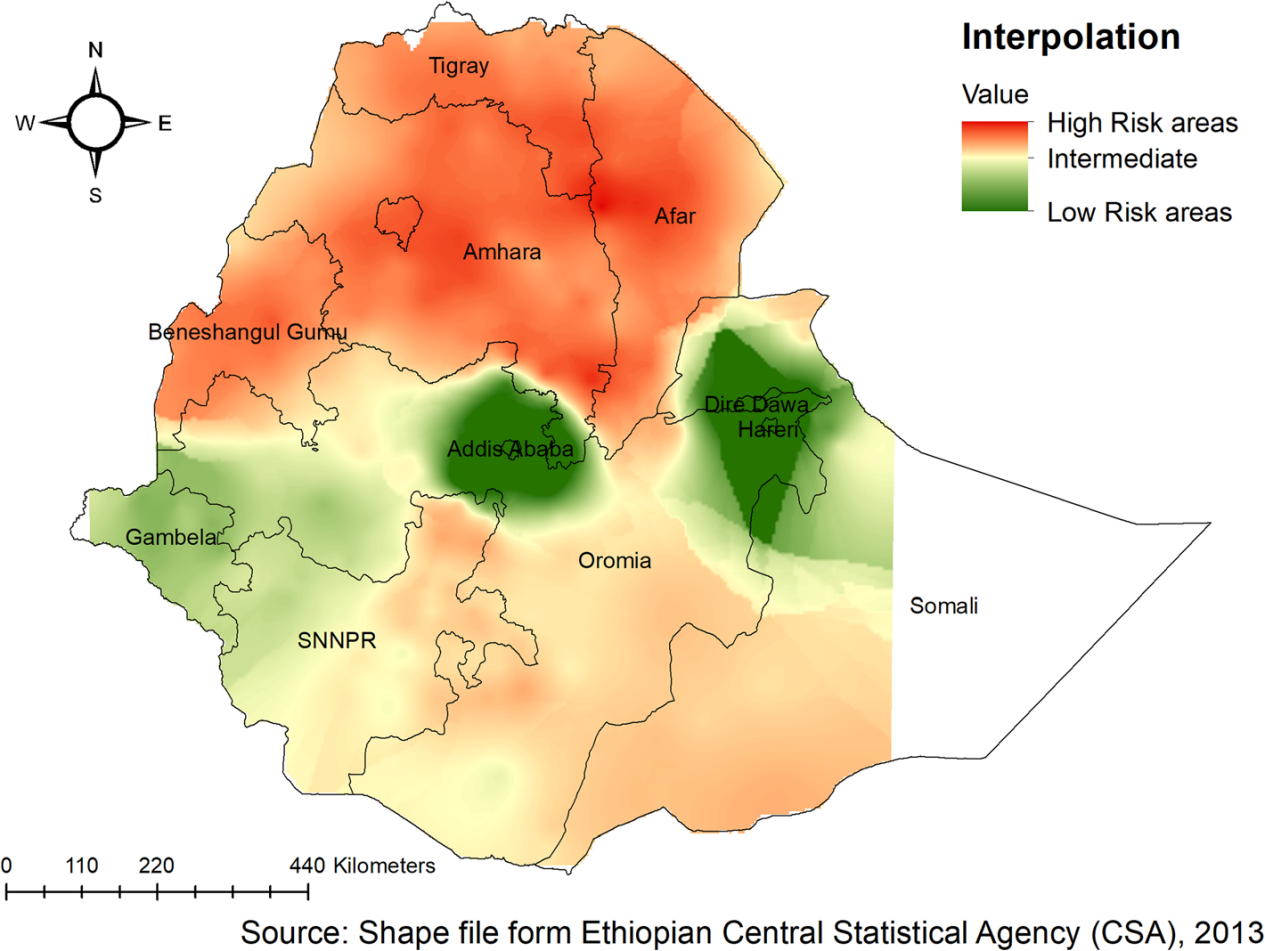
Ordinary Kriging interpolation of anthropometric failure in Ethiopia, EDHS 2011

**Fig. 10:**
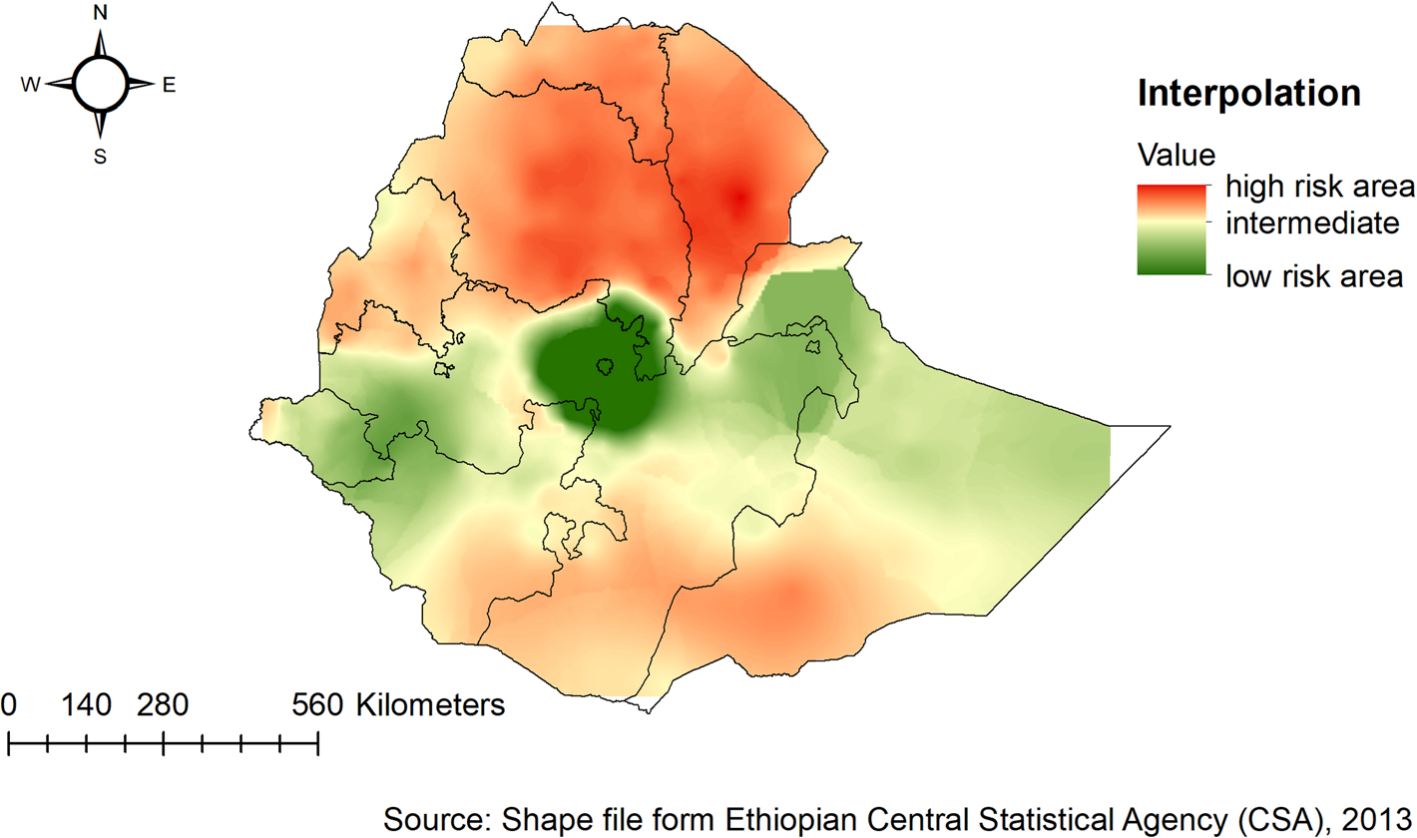
Ordinary Kriging interpolation of anthropometric failure in Ethiopia, EDHS 2016

The kriging interpolation analysis mapped the estimated distributions of anthropometric failures interpolating the available data to the areas where data were not taken. From EDHS 2005 sampled data, the geostatistical analysis predicts that the highest prevalence of childhood anthropometric failures was detected in the Amhara and southern parts of Tigray regions. While the predicted low anthropometric failures are located in Addis Ababa and the central part of Oromia (Fig. 8 ). In 2011, Kriging interpolation revealed that the highest predicted prevalence of anthropometric failures was found in Amhara, Tigray, Afar, and Benishangul Gumuz regions. In contrast, the predicted low anthropometric failures were detected in Addis Ababa, the central part of Oromia, Harari, and Dire Dawa (Fig. 9). Based on EDHS 2016, Kriging interpolation predict that the highest prevalence of anthropometric failures was detected in the Amhara, the southern and central part of Tigray, and Afar regions whereas, the lowest anthropometric failures were predicted in the Addis Ababa, central Oromia, and Gambella (Fig. 10).

### Multilevel analysis

Bi-variable multilevel logistic regression analysis was done to identify variables for multivariable multilevel logistic analysis and Variables with a p-value less than 0.2 were considered for multivariable analysis. In multivariable multilevel mixed-effect logistic regression analysis individual/household-level factors such as the age of the child, maternal BMI status, birth weight, initiation of breastfeeding, the number of under-five children in the house, maternal and paternal educational level were a significant predictor of childhood anthropometric failure.

Table 5 shows the results of the multivariable multilevel logistic regression analyses. At the individual-level variables, children above 5 months of age and children who started breastfeeding after an hour had a higher odd of anthropometric failure. On the other hand, the odds of having anthropometric failure for under-five-year-old children decreases by 25 % with an increase in birth weight (AOR = 1.25, 95 % CI: 1.16–1.49). Regarding household-level variables, children were more likely to had anthropometric failure if a mother had a low BMI (AOR = 1.35, 95 % CI: 1.01–1.95) relative to those children who had a mother with normal BMI. Moreover, having a mother with formal education significantly decreased the odds of having an anthropometric failure. Similarly, odds of having anthropometric failure were much lower among under-five children whose fathers are completed secondary education (AOR = 1.51, 95 % CI: 1.07–2.12).

**Table 5:** Factors associated with childhood anthropometric failures in Ethiopia by multilevel logistic regression analysis, EDHS 2016

Variables	Null model	Model 2 AOR(95 % CI)	Model 3 AOR(95 % CI)	Model 4 AOR(95 % CI)
**Individual level variables**				
**Child’s age(months )**				
0-5 m		1.00	---	1.00
6-11 m		2.52(3.22–5.11)	---	2.34[1.22–3.26]
12-23 m		4.47(5.00-7.44)	---	3.66[2.22–4.26]
24-35 m		4.22(5.00-7.25)	---	5.20[4.42–6.21]
Above 36 m		3.55(2.33–6.10)	---	4.12[3.11–5.22]
**Birth weight**				
Low < 2500		1.00	---	1.00
High > = 2500		1.40(1.05–1.89)	---	1.25[1.16–1.49]
**Initiation of breast feeding**				
Within an hour		1.00	---	1.32[1.16–1.54]
After an hour		1.42(1.12–1.56)	---	1.62[1.22–2.28]
After one day		1.33(1.1.88)	---	1.51[1.06–2.11]
**No of children under-five in the house hold**				
1		1.00	---	1.00
2		0.74(0.66–1.4)	---	0.89[0.69–1.03]
3		0.85(0.73–1.01)	---	0.96[0.79–1.12]
**Maternal BMI**				
Normal		1.00	---	1.00
Thin		1.22[1.01–2.2]	---	1.35[1.01–1.95]
Overweight		1.77[1.42–3.2]	---	0.79[0.52–0.95]
**Maternal educational level**				
No education		1.00	---	1.00
Primary		0.87[0.75–1.03]	---	0.82[0.66–0.96]
Secondary and above		0.66[0.36–1.03]	---	0.77[0.62–0.95]
**Paternal educational level**				
No education		1.00	---	1.00
Primary		0.92[0.88–1.22]	---	0.96[0.82–1.12]
Secondary and above		0.74[0.59–0.95]	---	0.75[0.46–0.89]
**Community level variables**				
**Residence**				
Urban		---	1.00	1.00
Rural		---	1.42[1.02–1.88]	1.38[1.07–2.21]
**Region**				
Dire Dawa		---	1.00	1.00
Tigray		---	5.35[4.65–8.01]	3.02[2.00-4.23]
Afar		---	1.45[1.05–2.01]	2.10[1.30–3.46]
Amhara		---	3.20[2.2–4.33]	4.65[3.22–5.35]
Oromiya		---	2.01[1.82–3.25]	2.01[1.52–3.15]
somali		---	3.05[2.11–5.02]	2.88[1.88–4.66]
Benishangul gumz		---	1.92[1.28–2.89]	1.96[1.42–2.84]
SNNP		---	1.23[1.00-1.67]	1.22[1.22–2.55]
Gambella		---	0.77[0.56–0.98]	0.67[0.37–0.95]
Harari		---	0.86[0.52–1.21]	0.71[0.46–1.20]
Addis Abeba		---	0.81[0.52-1.0.98]	0.57[0.37–0.89]
**Random effects**				
ICC%	25.0			
PCV%	1.00	52.23	70.24	74.93
MOR	11.82	4.43	3	1.92
**Model comparison**				
Log-likelihood ratio	-4879.62	-2555.6	-4277.59	-2366.74
AIC	9759.24	5111.2	8555.18	4733.48

NB: *CI * Confidence Interval, *AOR *Adjusted Odds Ratio, *AIC *Akakian Information Criteria, *MOR* median odds ratio

After fitting a multivariable multilevel logistic regression model on community-level factors, the multivariate results showed that community-level factors such as place of residence and region of residence were significantly associated with childhood anthropometric failure (Table 5). Children from rural households had 38 % higher odds (AOR = 1.38, 95 % CI: 1.07–2.21) of having anthropometric failure compared to children from urban households. Furthermore, the odds of having anthropometric failure were higher among under-five children in regions of (Tigray, AOR = 3.02; 95 % CI: 2.00–4.23; Afar, AOR = 2.10; 95 % CI 1.30–3.46; Amhara, AOR = 4.65; 95 % CI 3.22–5.35; Oromia, AOR = 2.01; 95 % CI: 1.52–3.15; Somali, AOR = 2.88, 95 % CI: 1.88–4.66; Benishangul Gumz, AOR = 1.96, 95 % CI: 1.42–2.84; SNNP, AOR = 1.22, 95 % CI: 1.02–2.55) relative to those who lived in more Dire Dawa. On the other hand, the odds of anthropometric failure were lower among children in Gambella and Addis Abeba regions as compared to Dire Dawa. However, no significant difference was apparent between children from the Dire Dawa and Harari regions.

## Discussion

In the present study, we identified spatial trends and determinants of anthropometric failure among under-five children over a decade, based on three consecutive DHS surveys in Ethiopia. A CIAF based classification was used for assessing the anthropometric status of under-five, with the advantages of identifying children with multiple conditions of under-nutrition in addition to children with wasting, stunting, and underweight [13, 26], which is neglected in most nutritional surveys. Our analysis shows how the conventional indices of stunting, wasting, and underweight, when used on their own, miss significant numbers of children who experience multiple anthropometric deficits, which is similar to studies conducted using CIAF [16, 18].

Our finding indicated that the trend of anthropometric failures decreased from 53.5 to 46.2 % from 2005 to 2016. This study revealed that 46.2 % [95 % CI: 45.5, 47.6] of children had anthropometric failures in the 2016 national survey. This is higher compared to the prevalence reported by conventional indices of stunting wasting and underweight in Ethiopia [11, 27]. Even though there was limited comparable studies similar to this anthropometric analysis using the CIAF model, the result of this study is in line with studies conducted in Ethiopia using 2014 mini demographic health survey 48.5 % [28] and Bangladesh 48.3 % [29], whereas higher than studies conducted in Tanzania 38.2 % and West Bengal of India 32.7 % [30]. On the other hand, lower than studies conducted in Yemen 70.1 [18] and southern India 58.59 % [14]. Ultimately, despite the progress that is made, the prevalence of anthropometric failure among children of 6–59 months of age continues to be a major public health problem in Ethiopia.

To date, few studies have explicitly examined the spatial disparity of anthropometric failures by taking into account the overall distribution of under-nutrition. The Moran’s *I* statistics suggested strong spatial dependence in variations on the levels of anthropometric failures across the regions in Ethiopia, this highlights that geography plays a substantial role in the levels of anthropometric failures in an area. Furthermore, a clear spatial pattern of childhood anthropometric failures was observed across the regions of Ethiopia over a decade. The anthropometric failures showed significant clustering in the districts belonging to the regions of Tigray, Amhara, Afar, Somali, and Benishangul Gumz. Overall, Tigray, Amhara, and Afar regions were among the higher risk regions of anthropometric failures across time. This could be because of high poverty rates in the above-mentioned areas/regions compared to other regions [6, 31, 32]. Besides, the culture and practice of child feeding might affect childhood nutritional status. Further, our analysis indicated that the observed variation in childhood anthropometric failures can be attributed to both individual and community-level factors. In the final model, individual and community-level factors accounted for about 74.93 % of the variation observed for childhood anthropometric failures.

This study indicated that children in the youngest age group of 0–5 months had a lower risk of anthropometric failure than children in the older age group. This finding was consistent with studies conducted in Tanzania [16] and Yemen [18]. This could be explained by the fact that children at their younger stage are getting a diet that is richer and complete or vulnerable to disease when a child grows old. The failure may result from the termination of breastfeeding, increment of nutritional demand during rapid physical and mental growth might it be the possible reasons.

The finding of this study indicated that children whose mothers are thin (having a BMI below 18.5) had more anthropometric failures as compared to children whose mothers had normal body weight (BMI 18.5-24.95). This finding is in line with other studies conducted in sub-Saharan Africa [16, 33] and India [17]. This group consists of more at-risk population segments for children anthropometric failure. Aside from the poor nutritional status of mothers, it leads to raising the infant’s risk of low birth weight and infant deficiency, thus, these conditions might lead to anthropometric failure.

We also found that childhood anthropometric failures are significantly associated with maternal and paternal education. Previous studies conducted have shown that maternal education has a positive effect in decreasing childhood anthropometric failure [16, 17]. The knowledge that mothers acquire from formal education could help them to adopt essential nutrition and hygiene behaviors that may prevent children anthropometric failures. Another reason might be that educated mothers have a better understanding of maternal and childcare education and information provided through media as compared to uneducated mothers which can help to avert anthropometric failures. Further paternal education was also positively associated with reducing childhood anthropometric failures [14]. Those children whose fathers had attended formal education had less chance to had anthropometric failures. Similar findings have been reported by other studies [16, 19]. Those fathers with formal education might be more knowledgeable on proper child feeding and hygiene practices, which have a positive effect to avert these failures. In Ethiopia lack of education is a widespread problem, which could be the possible factor for the prevention of anthropometric failures Ethiopia. Furthermore, the influence of education in improving the socio-economic ladder and reducing poverty level could have an impact on children’s nutritional status, as anthropometric failures are critical predictors of household capital.

There are some limitations of the current study that need to be considered in interoperating the results. Firstly, the conversion of CIAF outcomes into dichotomized childhood anthropometric status as an outcome variable may result in loss of information. Even if, the Analysis of the prevalence and spatial trends included three surveys while factors associated with CIAF were assessed only using the recent survey. Besides, the cross-sectional nature of the study prevents causality from being inferred between the independent and dependent variables. Furthermore, the DHS clusters with missing longitude and latitude data in the dataset were excluded from the spatial analysis which could affect the generalizability of the findings. Despite the above limitations, we believe that our findings and suggestions will contribute immensely to a better understanding of childhood malnutrition by providing insight into the overall undernutrition status. The representativeness of the data with a large sample size, as well as a nationally representative population-based study with a high response rate are strengths of this study that give high statistical power to infer the characteristics of the study population. Another important strength of this study is the use of multilevel logistic regression analysis, which was able to identify other factors beyond individual-level factors that would not be identified by using standard logistic regression analysis. The use of a combination of methods the spatial and regression statistics were also allowed cross-validation of the identified hotspots and cold spot areas.

## Conclusions

Findings from this study indicated that the prevalence of childhood anthropometric failures as measured by CIAF shows a significant decrease from the 2005 to 2016 surveys in Ethiopia. The spatial pattern of child anthropometric failures in Ethiopia was non-random among the three consecutive surveys with the global Moran’s I value of 0.494, 0.910, and 0.447 in EDHS 2005, 2011, and 2016 respectively. Our study revealed that children in Ethiopia, who had a lower wealth index, had an uneducated mother/father, had low birth weight, or had a mother with low BMI are at a higher risk of anthropometric failures. This study has underlined that the district-specific variation is substantial in modeling the risk of anthropometric failures. The identified socio-demographic characteristics and districts at an increased likelihood of anthropometric failure can inform localized intervention and prevention strategies to improve the nutritional status and healthcare of children in Ethiopia.

## Data Availability

Data we used for this study are publicly available in the MEASURE DHS program and you can access it from www.measuredhs.com after explaining the objectives of the study. Then after receiving the authorization letter, the data is accessible and freely downloaded.

## Abbreviations

AOR: Adjusted Odds Ratio
ANC: Antenatal Care
CSA: Central Statics Agency
CI: Confidence Interval
CIAF: Composite index of anthropometric failure
COR: Crude Odds Ratio
EDHS: Ethiopia Demographic and Health Survey
ICC: Intra Class Correlation Coefficient
LLR: Log-Likelihood Ratio
MOR: Median odds ratio
OR: Odds Ratio
RR: Relative Risk
PVC: Proportional Change in Variance
SNNPR: Southern Nations, Nationalities, and Peoples' Region
